# Eurothiocin A and B, Sulfur-Containing Benzofurans from a Soft Coral-Derived Fungus *Eurotium rubrum* SH-823

**DOI:** 10.3390/md12063669

**Published:** 2014-06-20

**Authors:** Zhaoming Liu, Guoping Xia, Senhua Chen, Yayue Liu, Hanxiang Li, Zhigang She

**Affiliations:** School of Chemistry and Chemical Engineering, Sun Yat-Sen University, Guangzhou 510275, China; E-Mails: liuzhaom@mail2.sysu.edu.cn (Z.L.); xiagp@mail2.sysu.edu.cn (G.X.); chensh65@mail2.sysu.edu.cn (S.C.); liuyayue@mail2.sysu.edu.cn (Y.L.)

**Keywords:** marine fungi, secondary metabolites, α-glucosidase inhibitor, theoretical calculations, ECD, TDDFT

## Abstract

Two new sulfur-containing benzofuran derivatives, eurothiocin A and B (**1** and **2**), along with five known compounds, zinniol (**3**), butyrolactone I (**4**), aspernolide D (**5**), vermistatin (**6**), and methoxyvermistatin (**7**), were isolated from the cultures of *Eurotium rubrum* SH-823, a fungus obtained from a *Sarcophyton* sp. soft coral collected from the South China Sea. The new compounds (**1** and **2**) share a methyl thiolester moiety, which is quite rare among natural secondary metabolites. The structures of these metabolites were assigned on the basis of detailed spectroscopic analysis. The absolute configurations of **1** and **2** were determined by comparison of the experimental and calculated electronic circular dichroism (ECD) data. Compounds **1** and **2** exhibited more potent inhibitory effects against α-glucosidase activity than the clinical α-glucosidase inhibitor acarbose. Further mechanistic analysis showed that both of them exhibited competitive inhibition characteristics.

## 1. Introduction

In the search for new pharmaceutical lead compounds, increasing attention is being given to marine fungi [1,2,3]. As marine fungi survive under harsh environmental conditions, it can be expected that they may have evolved to biosynthesize biologically interesting and chemically diverse compounds [3]. Many marine fungi are associated to marine invertebrates such as sponges and soft corals, and their secondary metabolites might contribute to protecting their hosts by chemically mediated defense mechanisms from dangers such as predation [4,5,6,7]. In some cases, there is evidence that associated marine microorganisms might be the true sources of bioactive metabolites originally isolated from their host organisms [8,9].

Among invertebrate-associated fungi, most studies of secondary metabolites have focused on those derived from sponges [3,10,11,12,13]. By contrast, although soft corals have been shown to host a variety of fungi [14,15,16,17], chemical investigation of these fungi are relatively rare. The few limited reports of soft coral-associated fungi provide a clue that they would be a promising source for structurally diverse and biologically active secondary metabolites [18,19,20,21,22]. Recently, in our ongoing search for new and potent natural products from marine fungi in the South China Sea [23,24,25,26,27], we initiated investigations of those fungi associated with corals. In the course of this study, a soft coral-derived fungus, *Eurotium rubrum* SH-823, attracted our attention because the EtOAc extract of the fungal fermentation on rice exhibited significant α-glucosidase inhibitory activity. α-glucosidase is an important target enzyme for the treatment of type-2 diabetes, which is a metabolic disorder characterized by elevated blood glucose [28,29]. Such reversible inhibitors, including acarbose and voglibose, are currently used clinically to control blood glucose levels of patients. To avoid or decrease the adverse effects of current agents and also to provide more candidates of drug choices, it is still necessary to search for new α-glucosidase inhibitors for further drug development. Chemical investigation of the bioactive extract led to the discovery of two new sulfur-containing benzofurans, namely, eurothiocin A (**1**) and B (**2**), along with five known compounds, zinniol (**3**), butyrolactone I (**4**), aspernolide D (**5**), vermistatin (**6**), and methoxyvermistatin (**7**) (Scheme I). The isolates were evaluated for their α-glucosidase inhibitory effects compared to the clinical drug acarbose. Details of the isolation, structure elucidation, and the results of α-glucosidase inhibition study of the isolated compounds are reported herein.

**Scheme I:**
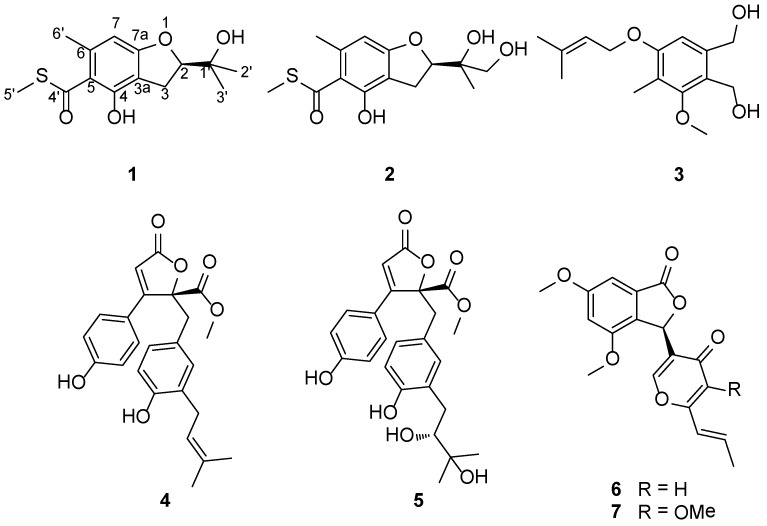
Chemical structures of compounds **1**–**7**.

## 2. Results and Discussion

Eurothiocin A (**1**) was obtained as a colorless oil. ESIMS data exhibited apparent molecular ions at 281.2 [M + H]^+^ and 283.2 [M + H + 2]^+^ in a 20:1 ratio, consistent with a compound containing a sulfur atom [30]. The molecular formula was subsequently determined as C_14_H_18_O_4_S on the basis of HREIMS (*m*/*z* 282.1621 [M]^+^, calcd 282.1618), indicating six degrees of unsaturation. The UV spectrum of **1** showed absorption maxima at 239 (sh) and 302 nm. The ^1^H spectrum in CDCl_3_ (Table 1) exhibited the presence of four singlet methyls (Me-2′, Me-3′, Me-5′, and Me-6′), one methylene (H_2_-3), one oxymethine (H-2), one aromatic proton (H-7), and one chelated phenolic hydroxyl group (4-OH). These findings were in agreement with the ^13^C NMR and DEPT data (Table 1), which exhibited 14 carbon signals as a carbonyl group (*δ*_C_ 197.7), two oxygenated sp^3^-hybridized carbons (*δ*_C_ 91.3 and *δ*_C_ 71.9), one methylene (*δ*_C_ 27.7), four methyls (*δ*_C_ 25.8, 23.8, 25.0, and 13.0), in addition to six sp^2^-hybridized carbons that were assigned to a pentasubstituted benzene ring. These functionalities, *i.e.*, one benzene ring and one carbonyl, account for five degrees of unsaturation, thus indicating one more ring being present in the molecule to satisfy the one remaining degree of unsaturation. 

**Table 1:** NMR spectroscopic data (CDCl_3_, 400/100 MHz) for **1** and **2** ^a^.

Position	1		2	
	*δ*_C_	*δ*_H_	*δ*_C_	*δ*_H_
2	91.3, C	4.71, dd (9.5, 8.4)	87.2, C	4.90, t (8.8)
3	27.7, CH_2_	3.05, dd (15.6, 8.4)	27.2, CH_2_	3.17, d (8.8)
		3.14, dd (15.6, 9.5)		
3a	111.5, C	-	111.3, C	-
4	158.2, C	-	158.2, C	-
5	116.1, C	-	116.2, C	-
6	141.8, C	-	141.9, C	-
7	105.7, CH	6.26, s	105.7, CH	6.24, s
7a	164.3, C	-	164.1, C	-
1'	71.9, C	-	73.7, C	-
2'	25.8, CH_3_	1.33, s	66.9, CH_2_	3.53, d (11.0)
				3.75, d (11.0)
3'	23.8, CH_3_	1.22, s	19.1, CH_3_	1.20, s
4'	197.7, C	-	197.8, C	-
5'	13.0, CH_3_	2.47, s	13.1, CH_3_	2.46, s
6'	25.0, CH_3_	2.69, s	25.1, CH_3_	2.68, s
4-OH	-	11.83, brs	-	11.83, brs

^a^ *δ* in ppm, *J* in Hz, TMS as internal standard.

**Figure 1:**
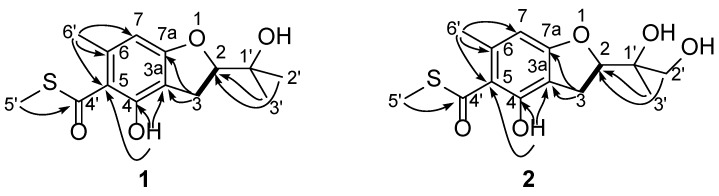
Selected ^1^H–^1^H COSY (bold line) and HMBC (arrow) correlations of compounds **1** and **2**.

Extensive analysis by 2D NMR, including HSQC, HMBC, and COSY, revealed the planar structure of eurothiocin A (**1**) as described below (Figure 1). In the ^1^H–^1^H COSY spectrum, the oxymethine proton H-2 was correlated to the methylene protons H_2_-3, revealing the connectivity of C-2 to C-3. HMBC cross-peaks from H_2_-3 to C-3a, C-7a, and C-4 and from H-2 to C-3a and C-7a established the dihydrobenzofuran ring with connectivity of C-2 to C-7a via an oxygen atom. Further correlations from *gem*-dimethyl groups (H_3_-2′ and H_3_-3′) to C-1′ and C-2 indicated that the oxygenated sp^3^ quaternary carbon C-1′ was attached to C-2′, C-3′, and C-2. The aromatic methyl signal H_3_-6′ (*δ*_H_ 2.69) exhibited HMBC correlations to C-5, C-6, and C-7, establishing the location of this methyl group. An additional four-bond W-type correlation from H_3_-6′ to the carbonyl carbon C-4′ connected C-4′ to C-5. The phenolic hydroxyl 4-OH was placed *ortho* to the carbonyl substituent C-4′ on the basis of its ^1^H NMR chemical shift (*δ*_H_ 11.83), as well as the HMBC correlations of its proton to C-3a, C-4, and C-5. Lastly, the presence of a methyl thioester was revealed by HMBC correlations of the *S*-CH_3_ methyl protons (*δ*_H_ 2.47) to the carbonyl carbon C-4′ (*δ*_C_ 197.7), and the ^1^H and ^13^C NMR chemical shifts for *S*-CH_3_ (*δ*_H_/*δ*_C_ 2.47/13.0) which are characteristic of a methyl thiolester group [31,32]. Moreover, the base peak in the EIMS spectrum corresponds to loss of 47 mass units, which is consistent with the expected [M − SCH_3_]^+^ fragmentation peak [32]. These data led to the assignment of the structure of eurothiocin A (**1**) as shown.

**Figure 2:**
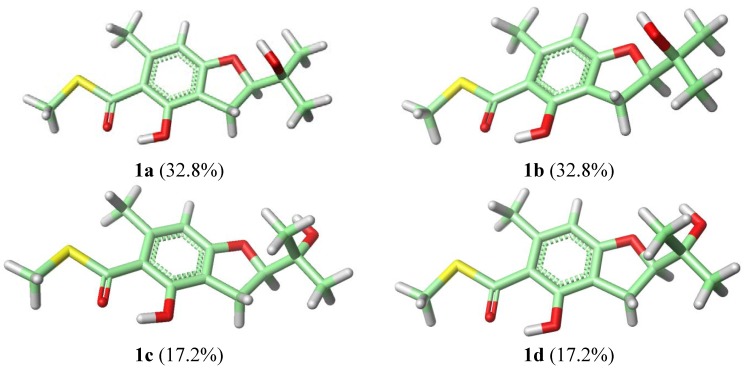
Most stable conformers of (*R*)-**1** calculated at the DFT/CAM-B3LYP/aug-cc-pVDZ level of theory. Relative populations are in parentheses.

**Figure 3:**
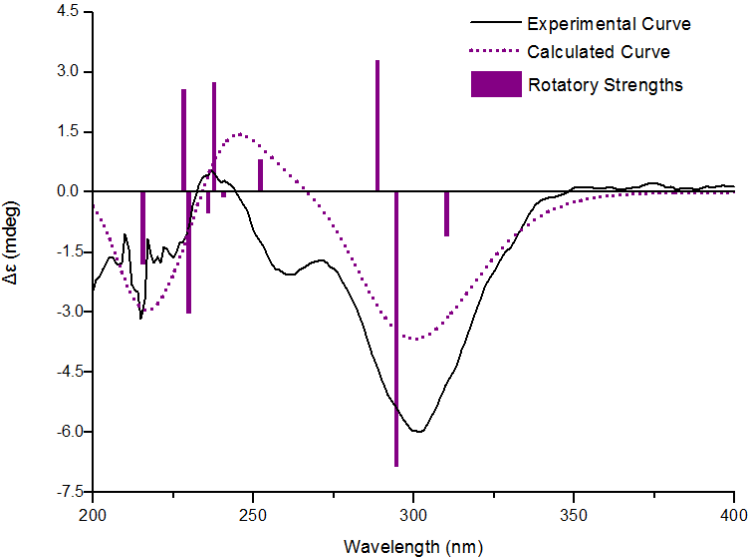
Experimental and calculated electronic circular dichroism (ECD) spectra of **1** ^a^*.*

^a^ Black solid line: ECD spectrum of **1** in MeCN solution. Purple dotted line: calculated ECD spectrum for (*R*)-**1** with CAM-B3LYP/aug-cc-pVDZ method on DFT-optimized structure. Purple bars: rotational strength values of the lowest-energy conformer of (*R*)-**1** optimized with CAM-B3LYP/aug-cc-pVDZ.

To assign the absolute configuration of **1**, ECD spectrum was measured in MeCN solution and compared with those calculated by quantum-mechanics. The conformational analyses of (*R*)-**1** were carried out with molecular mechanics (using the Merck molecular force field, MMFF) based on a Monte Carlo algorithm. Eight conformers with relative energy within 1 kcal/mol were generated and further geometry-optimized at CAM-B3LYP/aug-cc-pVDZ level using Gaussian 03 program. Four stable conformers were obtained (Figure 2), and frequency analyses were performed at the same level to show no imaginary frequencies. ECD spectra for the four conformers were calculated using TD-DFT method at CAM-B3LYP/aug-cc-pVDZ level. The final Boltzmann factor-weighted theoretical ECD spectrum (Figure 3 and Supplementary Figure S15) was similar to the experimental ECD spectrum, which showed a positive Cotton effect at 241 nm and a strong negative Cotton effect at 302 nm, respectively. Thus, the absolute configuration of compound **1** was established as *R*.

Eurothiocin B (**2**) was isolated as a white amorphous solid. Its HREIMS spectrum displayed a molecular ion peak at *m*/*z* [M]^+^ 298.0942 (calcd 298.0941), corresponding to the molecular formula C_14_H_18_O_5_S. The ^1^H and ^13^C NMR spectra together with HSQC correlations for eurothiocin B (**2**) showed one carbonyl carbon (C-4′, *δ*_C_ 197.8), six sp^2^-hybridized quaternary (one protoned), two methylenes (C-3 and C-2′, one of which was oxygented), one oxymethine (C-2), one oxygenated quarternary carbon (C-1′), and three methyls (Me-3′, Me-5′, and Me-6′). A detailed comparison of the NMR data with those for eurothiocin A (**1**) revealed that **2** differed from **1** only at C-2′. The methyl signal at *δ*_H_ 1.33 (s, H-2′) and *δ*_C_ 25.8 (C-2′) observed in the spectrum of **1** were replaced by oxygenated methylene protons at *δ*_H_ 3.53 and 3.75 (both d, *J* = 11.0 Hz) and *δ*_C_ 66.9 (C-2′) in the NMR spectrum of **2**, which suggested that **2** is a 2′-oxygenated derivative of **1**. HMBC correlations from H_3_-3′ to C-1′, C-2′, and C-2, from H_2_-2′ to C-1′, C-3′, and C-2, and from H-2 to C-1′, C-2′ and C-3′, further supported the proposed planar structure for **2**.

**Figure 4:**
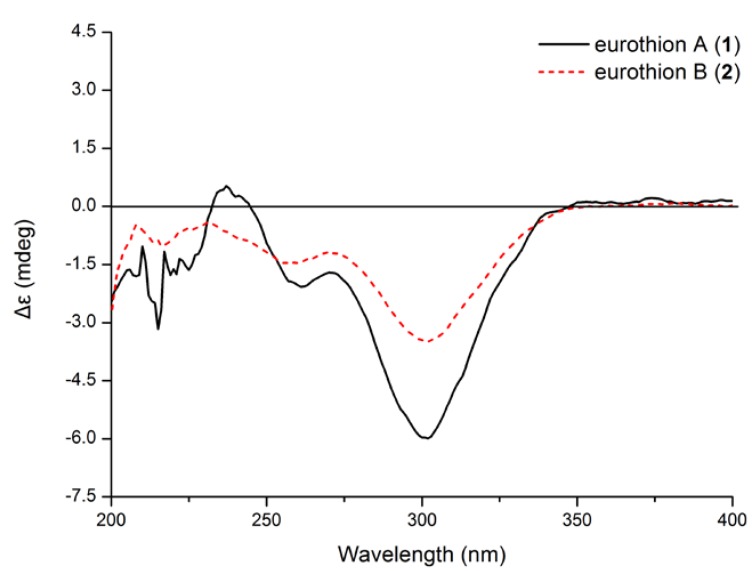
Comparison of experimental ECD spectra of eurothion A (**1**) and B (**2**).

According to comparison of the CD spectrum with that of **1** and their structural similarities (Figure 4), the absolute configuration at C-2 of eurothiocin B (**2**) is proposed to be the same as in eurothiocin A (**1**), but the stereochemistry at C-1′ of the side chain is unknown. Consequently, the overall absolute configuration of eurothiocin B (**2**) remains to be determined.

The structures of the known compounds were identified as zinniol (**3**) [33], butyrolactone I (**4**) [34], aspernolide D (**5**) [35], vermistatin (**6**) [36,37], and methoxyvermistatin (**7**) [37], by comparison of their spectroscopic data and optical rotations with those reported in the literature.

**Figure 5:**
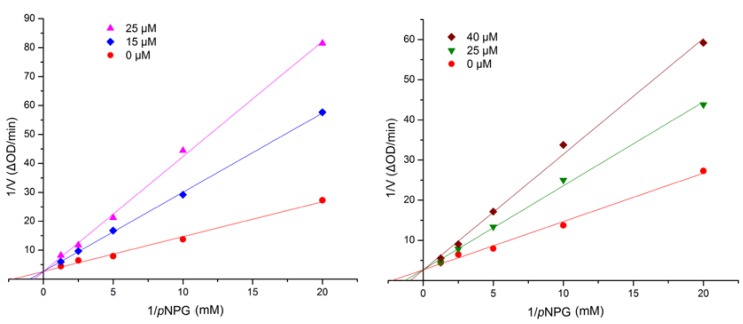
Kinetic analysis of the inhibition of α-glucosidase by compounds **1** (**left**) and **2** (**right**). More details are in the Supplementary Information.

The α-glucosidase inhibitory effects of the isolates were evaluated along with the clinical α-glucosidase inhibitor acarbose (positive control). As a result (Table 2), the compounds **1** and **2** were the most active, and showed better inhibitory potential (IC_50_ = 17.1 and 42.6 μM, respectively) than acarbose (IC_50_ = 376.7 μM). In order to examine the type of inhibition of the new compounds **1** and **2**, further kinetic studies were carried out by the Lineweaver-Burk plot method. The results are shown in Figure 5, indicating that **1** and **2** are competitive inhibitors of α-glucosidase. More details are in the Supplementary Information.

**Table 2:** α-Glucosidase Inhibitory Activities ^a^.

Compounds	1	2	4	5	6	7	Acarbose ^b^
IC_50_ (μM)	17.1 ± 0.7	42.6 ± 1.4	98.5 ± 3.3	110.8 ± 1.7	107.1 ± 2.6	236 ± 4.2	376.7 ± 5.2

^a^ IC_50_ values are shown as mean ± SD from two independent experiments. The inhibitory activity of compound **3** was not tested due to the limited amount; ^b^ Positive control.

## 3. Experimental Section

### 3.1. General

Melting points were measured on an X-4 micromeltin-point apparatus (Cany Precision Instruments Co., Ltd., Shanghai, China) and are uncorrected. Optical rotations were recorded with an MCP 300 (Anton Paar, Shanghai, China) polarimeter at 25 °C. UV data were measured on a UV-240 spectrophotometer (Shimadzu, Beijing, China). CD data were recorded with a J-810 spectropolarimeter (JASCO, Tokyo, Japan). The NMR data were recorded on a Bruker Avance 400 spectrometer (Bruker, Beijing, China) at 400 MHz for ^1^H and 100 MHz for ^13^C in CDCl_3_, respectively. All chemical shifts (δ) are given in ppm with reference to TMS, and coupling constants (J) are given in Hz. LRESIMS spectra were recorded on a Finnigan LCQ-DECA mass spectrometer (Finnigan, Beijing, China). EIMS on a DSQ EI-mass spectrometer (Thermo, Shanghai, China) and HREIMS data were measured on a MAT95XP high-resolution mass spectrometer (Thermo). Column chromatography (CC) was performed on silica gel (200–300 mesh, Qingdao Marine Chemical Factory, Qingdao, China) and Sephadex LH-20 (Amersham Pharmacia, Piscataway, NJ, USA). Precoated silica gel plates (Qingdao Huang Hai Chemical Group Co., Qingdao, China; G60, F-254) were used for thin layer chromatography. Semipreparative HPLC was performed on a Waters Breeze HPLC system using a Phenomenex Luna (Phenomenex, Torrance, CA, USA) C_18_ column (250 × 10 mm, 5 μm), flow rate, 2.0 mL/min.

### 3.2. Fungal Material

The fungal strain used in this study was isolated from a piece of fresh tissue from the inner part of the soft coral *Sarcophyton* sp., which was collected from Xuwen National Coral Reef Nature Reserve in the South China Sea in September 2012. It was obtained using the standard protocol for the isolation of endophytic microbes. This isolate was identified by Hanxiang Li and assigned the accession number SH-823. A voucher strain was deposited in School of Chemistry and Chemical Engineering, Sun Yat-sen University, Guangzhou, China.

### 3.3. Extraction and Isolation

The fungus *E. rubrum* SH-823 was fermented on autoclaved rice solid-substrate medium (twenty 500 mL Erlenmeyer flasks, each containing 50 g of rice and 50 mL of distilled water) for 30 days at 25 °C. Following incubation, the mycelia and solid rice medium were extracted with EtOAc. The organic solvent was filtered and concentrated under reduced pressure to yield 4.7 g of organic extract. The extract was subjected to silica gel CC using gradient elution with petroleum ether-EtOAc from 90:10 to 0:100 (*v*/*v*) to give twelve fractions (Frs.1–12). Fr. 4 (517 mg) was further purified by silica gel CC using 30% EtOAc-light petroleum to afford seven subfractions (Frs.4.1–4.7). Fr.4.3 (35 mg) was applied to Sephadex LH-20 CC, eluted with CHCl_3_/MeOH (1:1), to obtain eight subfractions (Frs.4.3.1–F4.3.8). Fr.4.3.4 (14 mg) was further purified by RP-HPLC (70% MeOH in H_2_O for 5 min, followed by 70%–100% over 25 min; 2.0 mL/min) to afford **1** (2.7 mg, *t*_R_ 16.5 min) and **2** (1.6 mg, *t*_R_ 23.0 min).

**Compound 1**: Colorless oil; 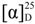 −135 (*c* 0.20, MeCN); UV (MeOH) (*λ*_max_) (log *ε*) 239 (sh), 302 (3.97) nm; ^1^H and ^13^C NMR spectroscopic data, see Table 1; EIMS *m*/z 282 [M]^+^, HREIMS *m*/*z* 282.1621 ([M]^+^, C_14_H_18_O_4_S, calcd 282.1618).

**Compound 2**: White amorphous power (CHCl_3_); m.p. 122–123 °C; 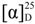 −69 (*c* 0.29, MeCN); UV (MeOH) (*λ*_max_) (log *ε*) 243 (sh), 301 (3.95) nm; ^1^H and ^13^C NMR spectroscopic data, see Table 1; EIMS *m*/*z* 298 [M]^+^, HREIMS *m*/*z* 298.0942 [M]^+^ (C_14_H_18_O_5_S, calcd 298.0941).

### 3.4. Calculation of ECD Spectra

Molecular mechanics calculations were run with Spartan '10 (Wavefunction, Inc., Irvine, CA, USA) with standard parameters and convergence criteria. DFT and TDDFT calculations were run with Gaussian 03 (Gaussian, Wallingford, CT, USA) with default grids and convergence criteria. TDDFT calculations were carried out by using CAM-B3LYP/aug-cc-pVDZ method and included 10 single excited states in each case. The IEF-PCM solvent model for MeCN was included in all cases. ECD spectra were generated using the program SpecDis 1.6 (University of Würzburg, Würzburg, Germany) and OriginPro 8.5 (OriginLab, Ltd., Northampton, MA, USA) from dipole-length rotational strengths by applying Gaussian band shapes with sigma = 0.40 ev and UV shift = +24 nm. All calculations were performed with High-Performance Grid Computing Platform of Sun Yat-Sen University.

### 3.5. Assay for α-Glucosidase Inhibitory Activity

α-*Glucosidase* was assayed according to standard procedures [38,39] by following the hydrolysis of nitrophenyl glycosides by monitoring formation of *p*-nitrophenol spectrometrically at 400 nm. The reaction mixture (final volume, 1 mL) consisted of the enzyme solution (20 μL, Sigma 9001-42-7, E.C 3.2.1.20), substrate (10 mM *p*-nitrophenyl-α-glucopyranoside, 20 μL, Fluka, BioChemika, Buchs, Switzerland) in 50 mM potassium phosphate buffer (pH 7.0) and test sample dissolved in DMSO (20 μL). The inhibitors were pre-incubated with the enzyme at 37 °C for 30 min, and the substrate was then added. The reaction was monitored spectrophotometrically by measuring the absorbance at 400 nm. Acarbose was used as a positive control. The inhibitory activity of test compound was determined as a percentage in comparison to a blank according with the following equation: The inhibition rates (%) = [(OD_control_ − OD_control blank_) − (OD_test_ − OD_test blank_)]/(OD_control_ − OD_control blank_) × 100%. The IC_50_ values of compounds were calculated by the nonlinear regression analysis and expressed as the mean ± SD of two distinct experiments. Kinetic parameters were determined using the Lineweaver-Burk double-reciprocal plot method at increasing concentration of substrates and inhibitors.

## 4. Conclusions

Chemical investigation of a soft coral-derived fungus *Eurotium rubrum* SH-823 led to the discovery of two new sulfur-containing benzofurans, eurothiocin A (**1**) and B (**2**), together with five known compounds, zinniol (**3**), butyrolactone I (**4**), aspernolide D (**5**), vermistatin (**6**), and methoxyvermistatin (**7**). To the best of our knowledge, the presence of a methyl thiolester group is quite rare among secondary metabolites. Two examples resembling compounds **1** and **2** are mortivinacin [31] and resorthiomycin [40,41], which were isolated from *Mortierella vinacea* and *Streptomyces collinus*, respectively. The isolated compounds were evaluated for their α-glucosidase inhibitory effects. Compounds **1** and **2** exhibited more potent inhibitory effects against α-glucosidase activity than the clinical α-glucosidase inhibitor acarbose. Further mechanistic analysis showed that the two compounds exhibited competitive inhibition characteristics.

## Supplementary Files

Supplementary File 1 Supplementary Information (PDF, 1342 KB)
