# Supplementary material for: Eurothiocin A and B, Sulfur-Containing Benzofurans from a Soft Coral-Derived Fungus Eurotium rubrum SH-823
Source: Mar Drugs. 2014 Jun 20;12(6):3669–80. doi: 10.3390/md12063669 (PMC4071596; doi:10.3390/md12063669)
Supplement: Supplementary File 1 — Supplementary Information (PDF, 1342 KB) [file marinedrugs-12-03669-s001.pdf]

Supplementary Information

Figure S1. ^{1}H NMR spectrum (400 MHz, CDCl3) of eurothiocin A (**1**).

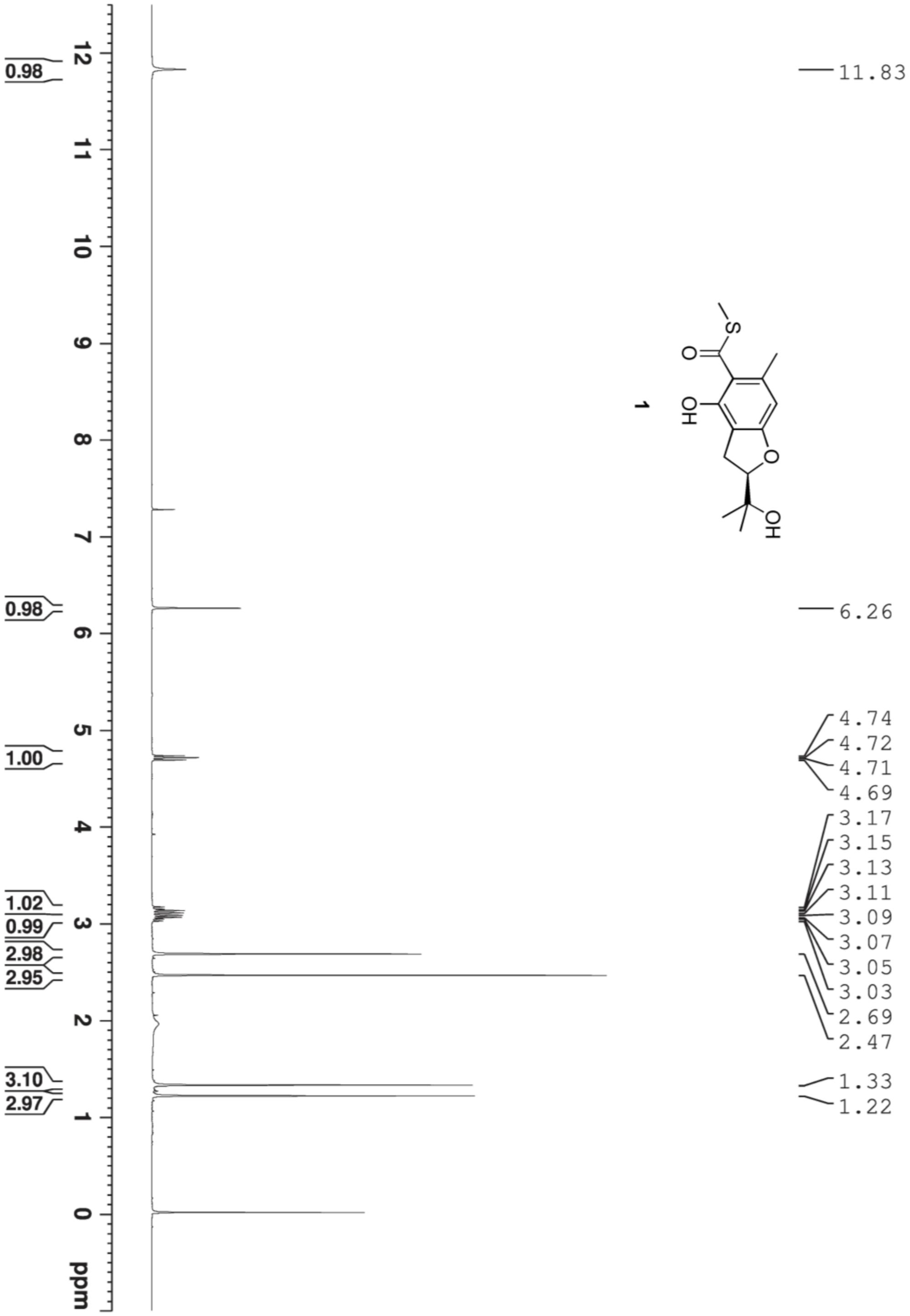

Figure S2. ^{13}C NMR spectrum (100 MHz, $CDCl_3$) of eurothiocin A (**1**).

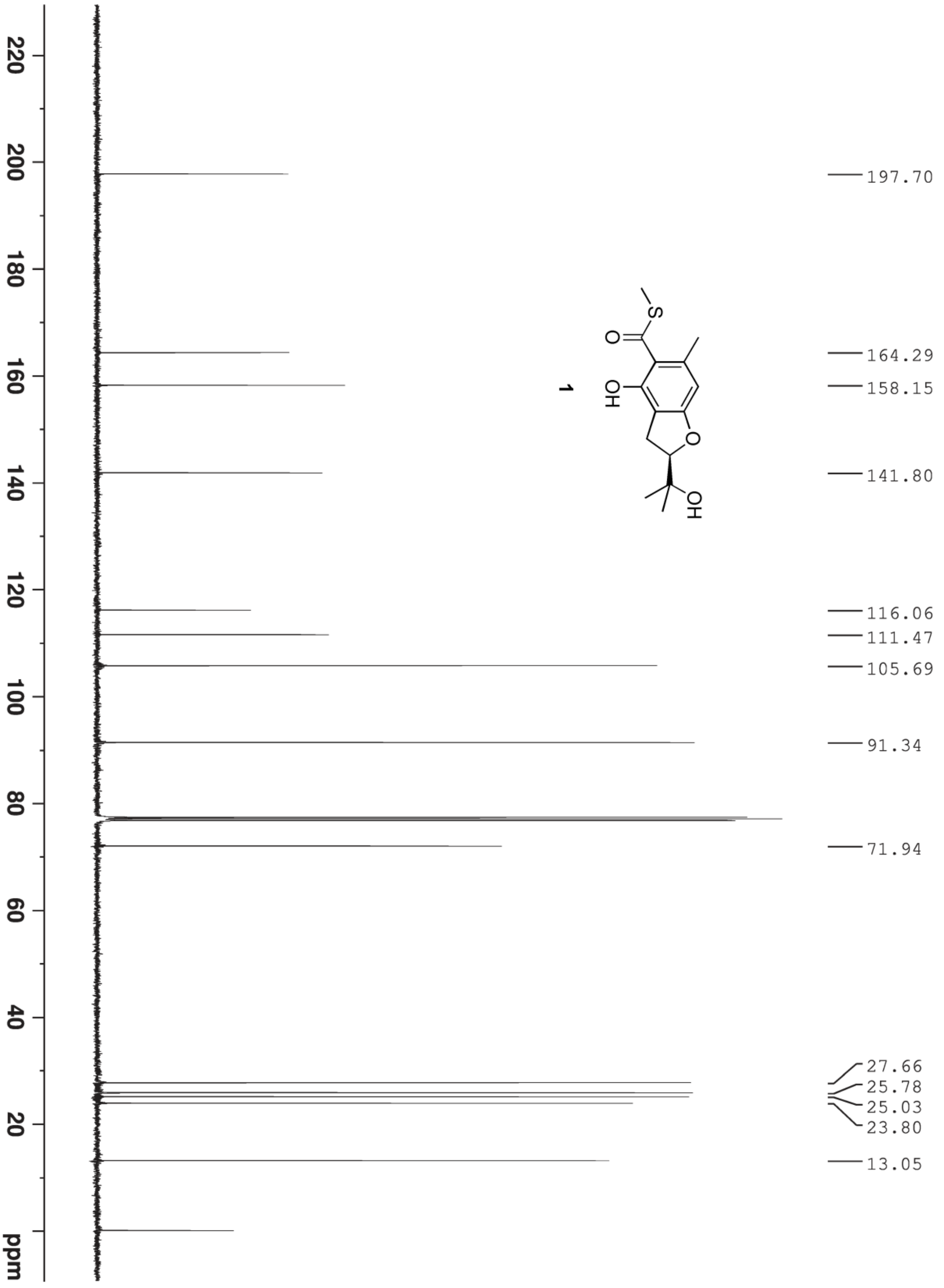

Figure S3. DEPT 90 spectrum of eurothiocin A (**1**).

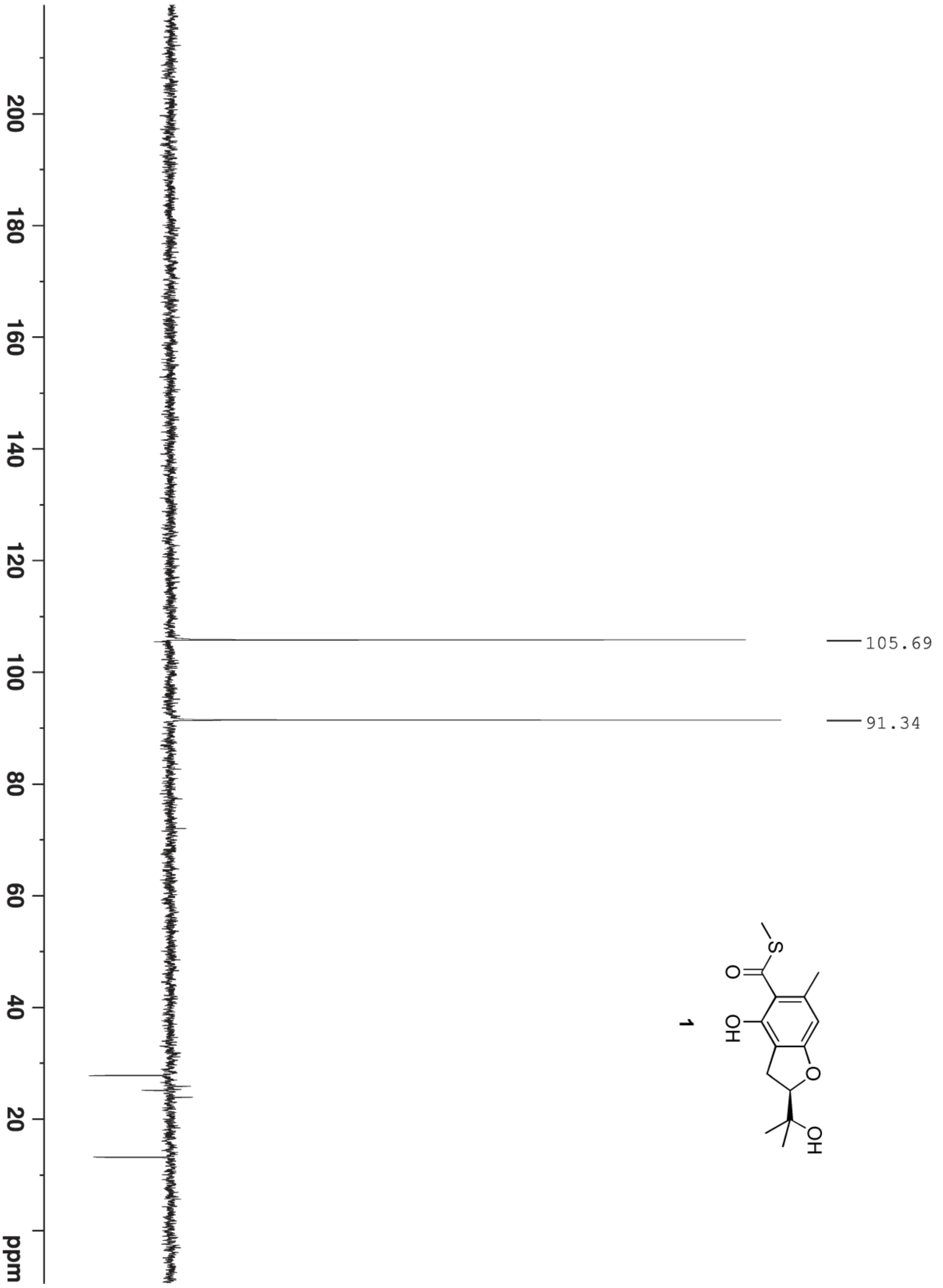

Figure S4. DEPT 135 spectrum of eurothiocin A (**1**).

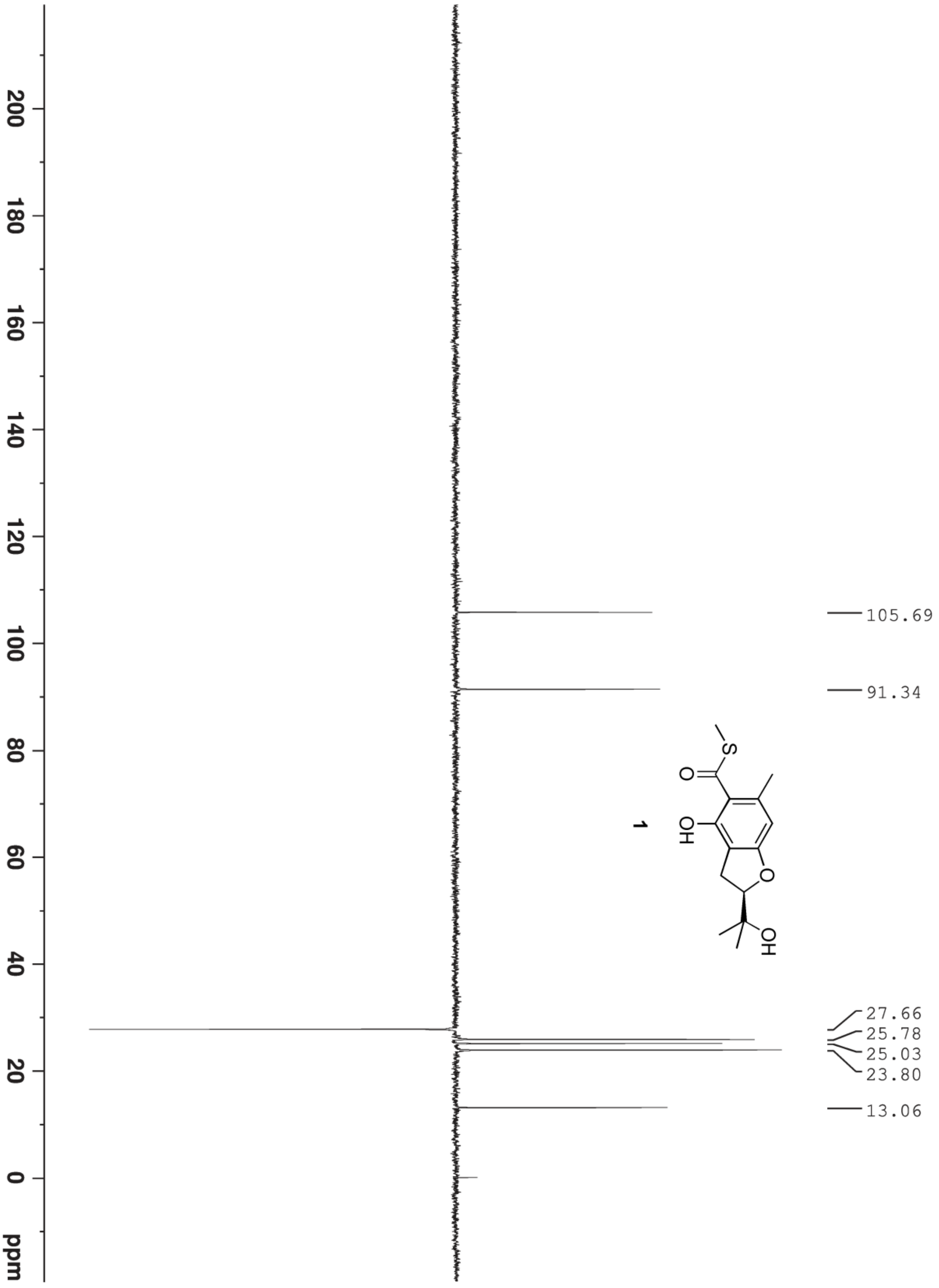

Figure S5. H–H COSY spectrum of eurothiocin A (**1**).

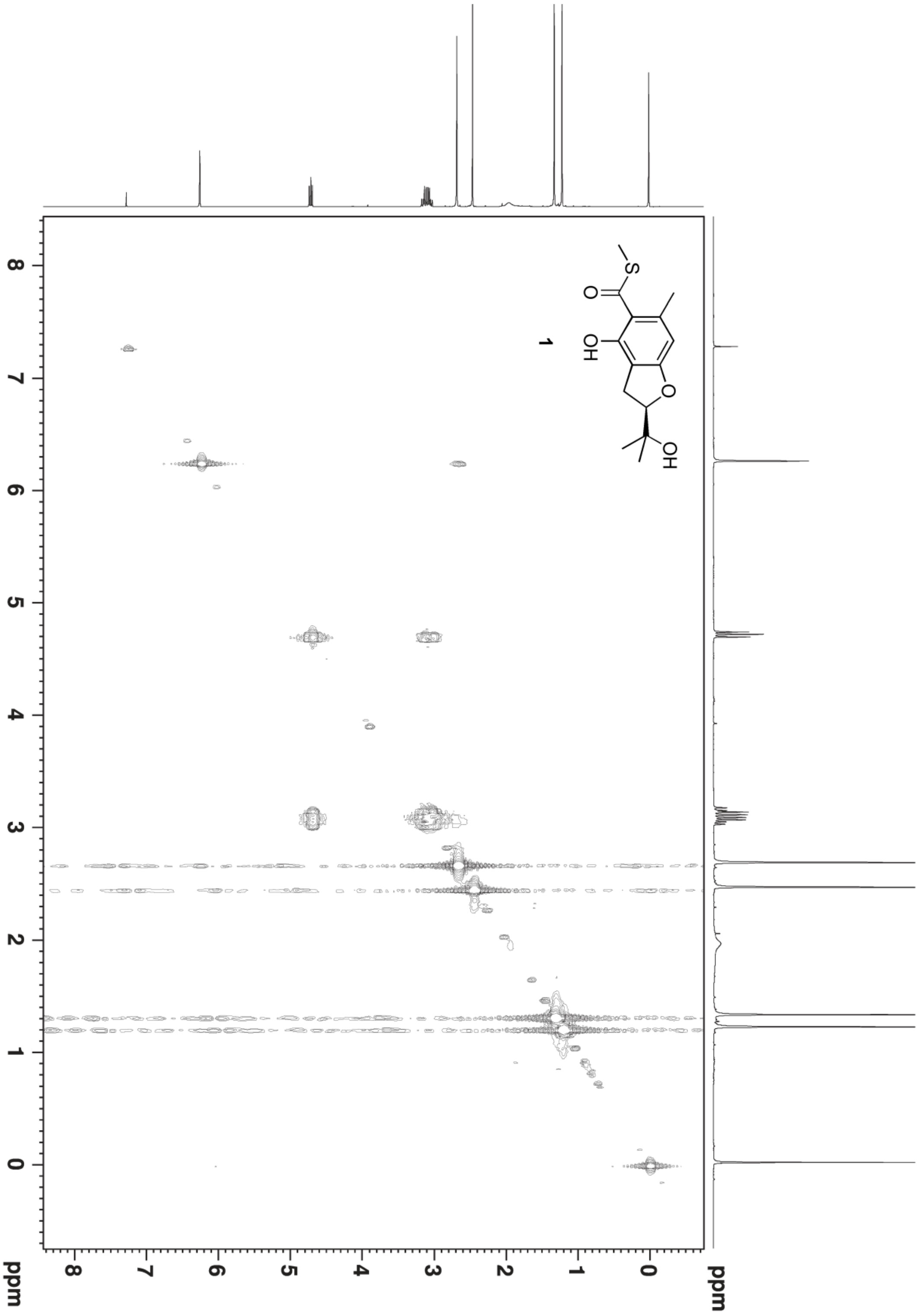

Figure S6. HSQC spectrum of eurothiocin A (**1**).

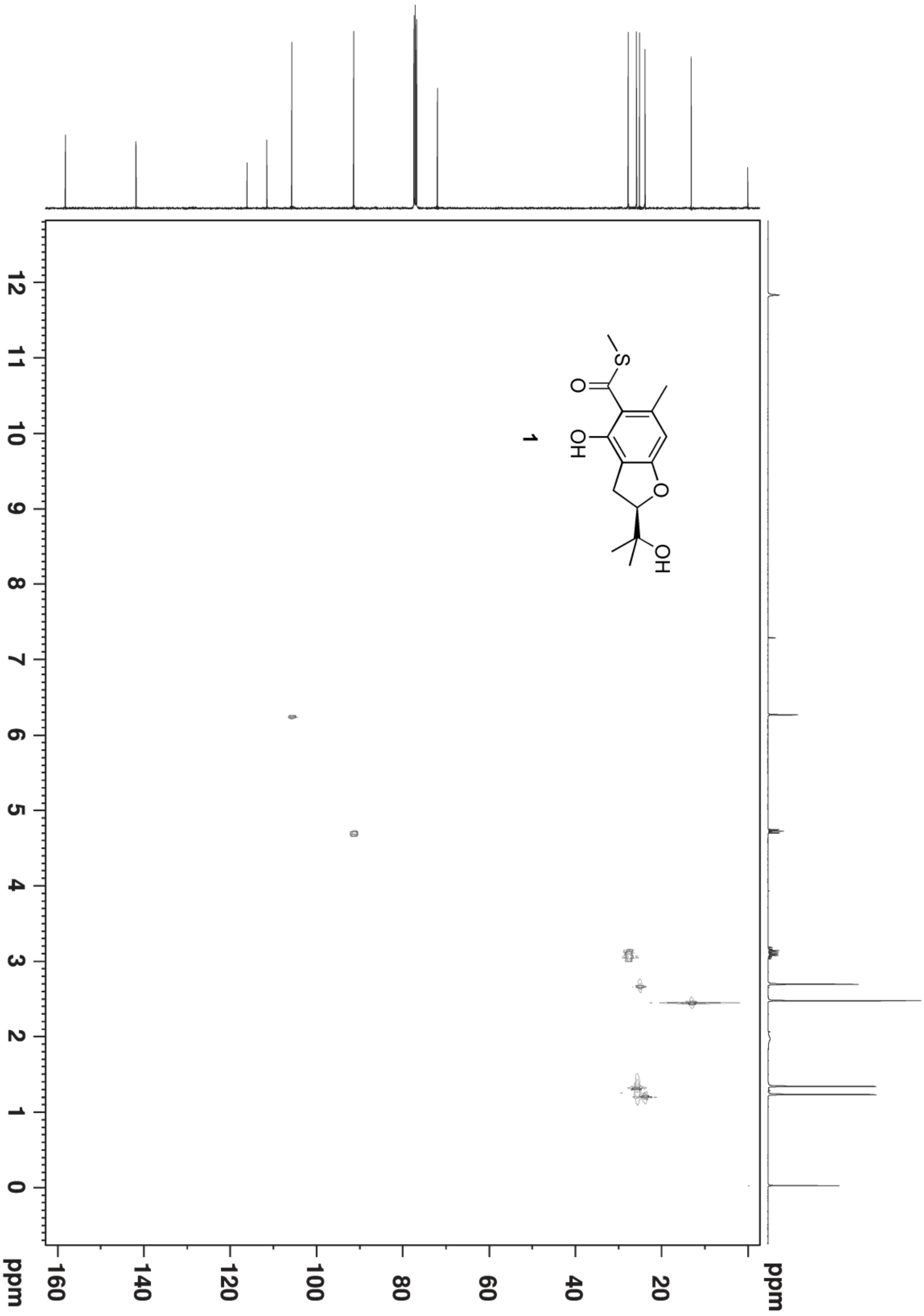

Figure S7. HMBC spectrum of eurothiocin A (**1**).

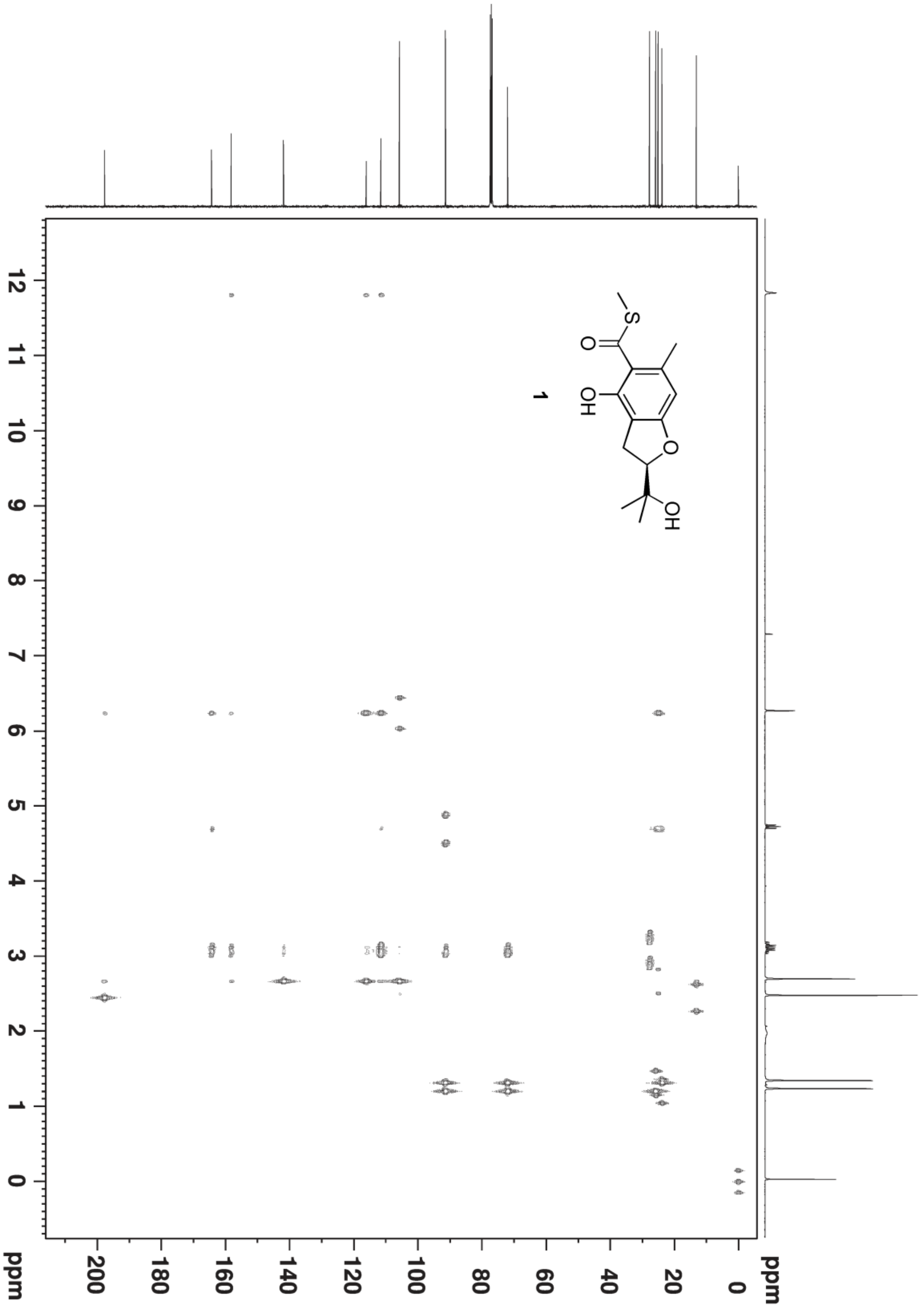

Figure S8. ^{1}H NMR spectrum (400 MHz, $CDCl_3$) of eurothiocin B (**2**).

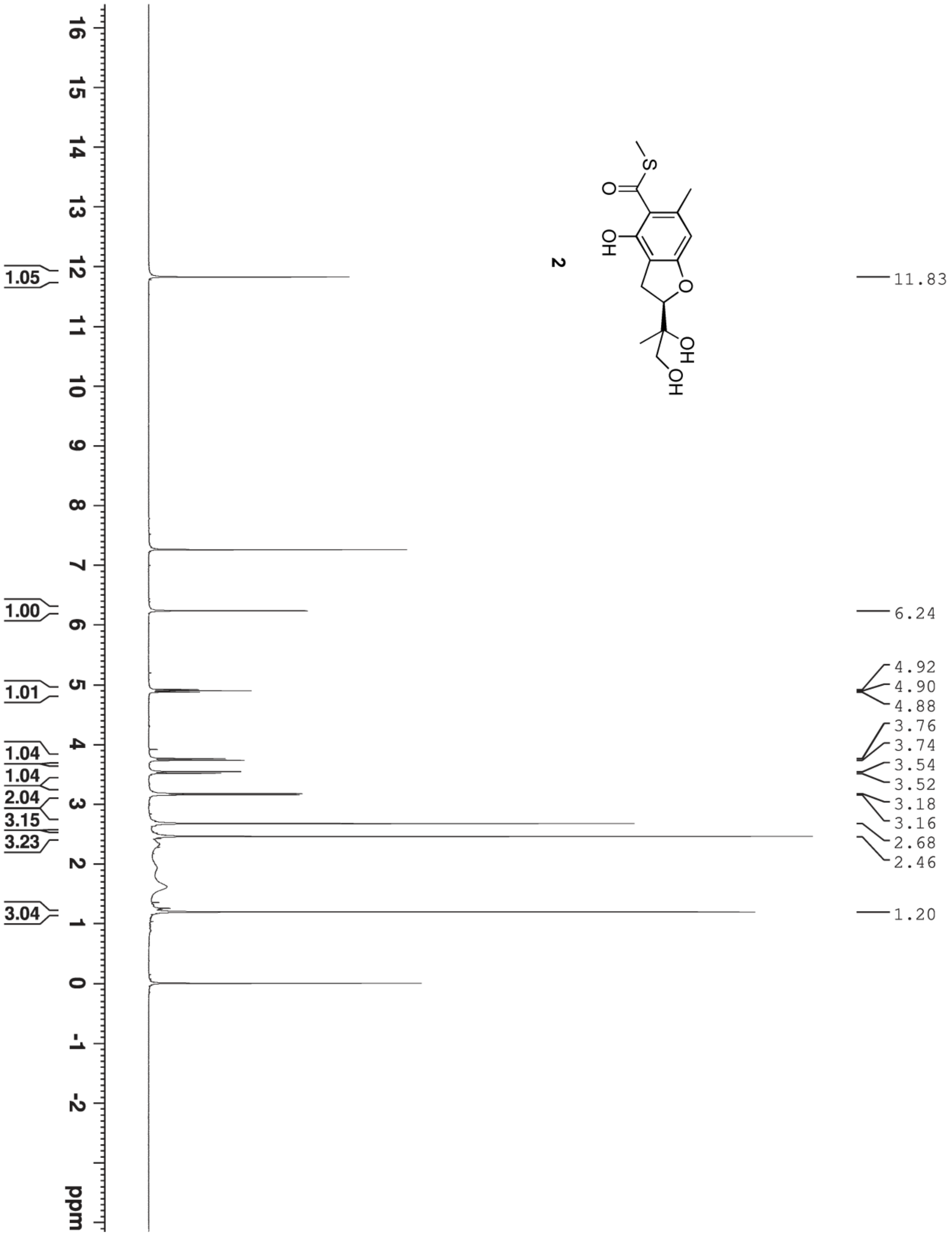

Figure S9. ^{13}C NMR spectrum (100 MHz, $CDCl_3$) of eurothiocin B (**2**).

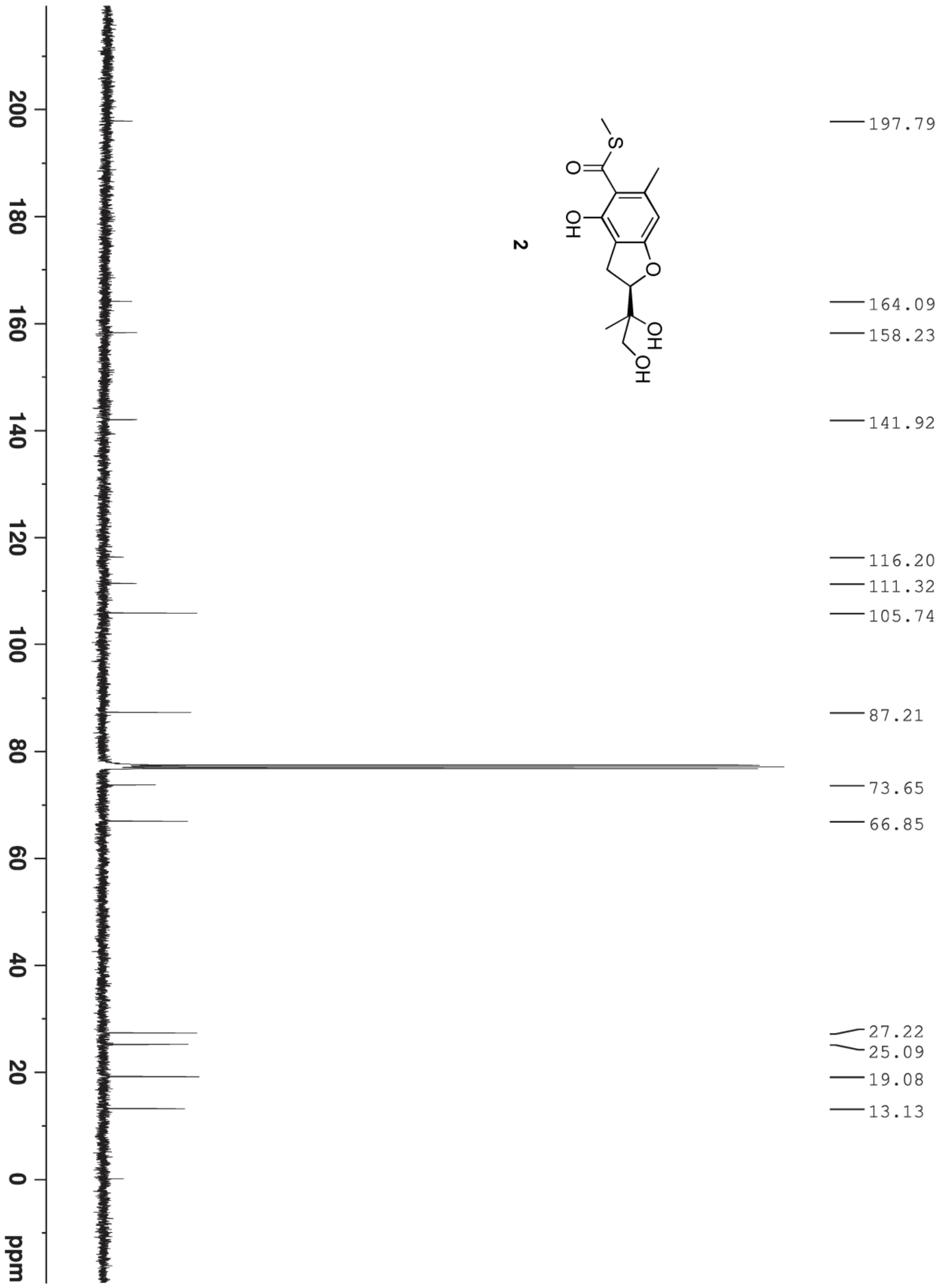

Figure S10. DEPT 90 spectrum of eurothiocin B (**2**).

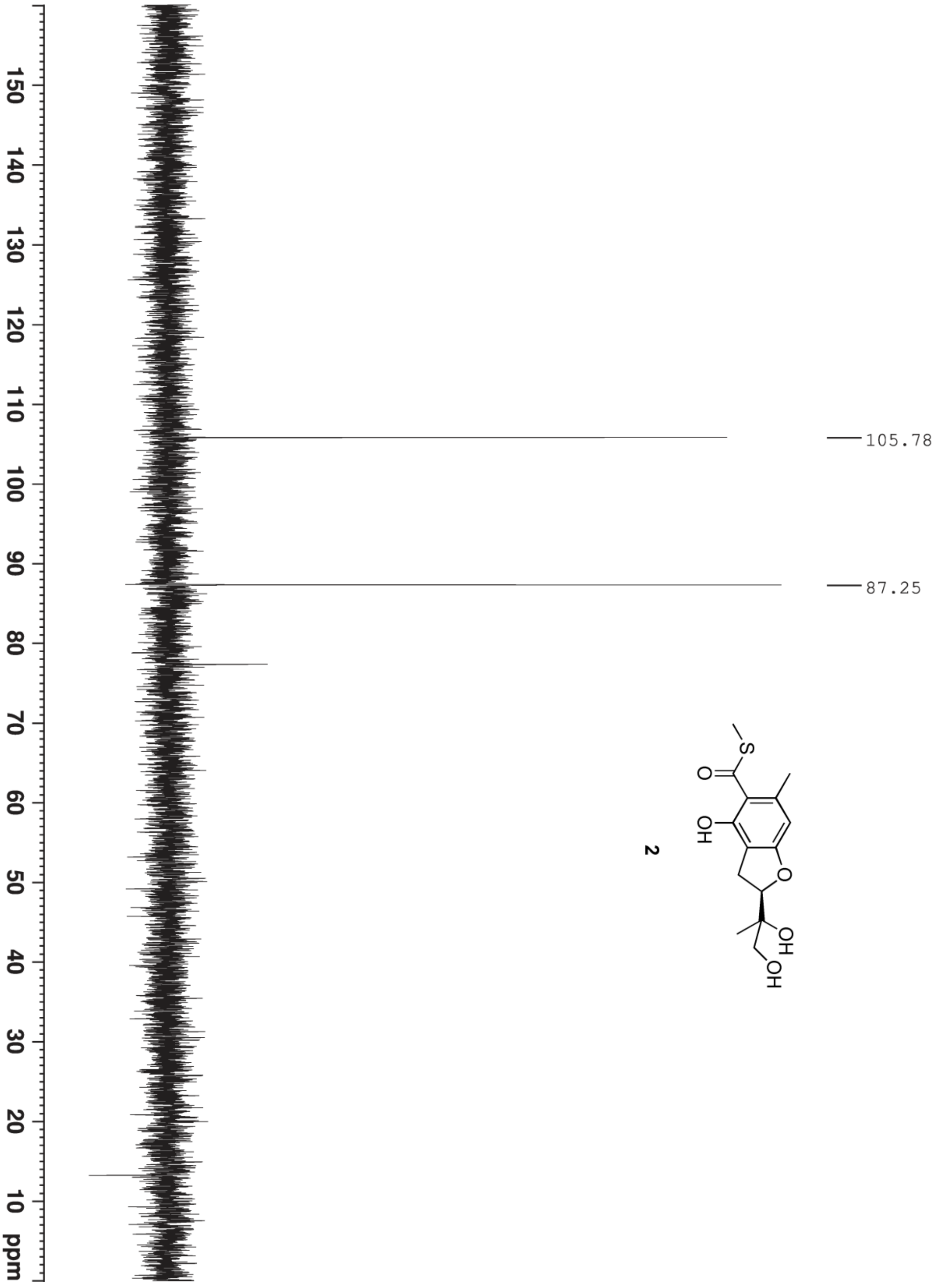

Figure S11. DEPT 135 spectrum of eurothiocin B (**2**).

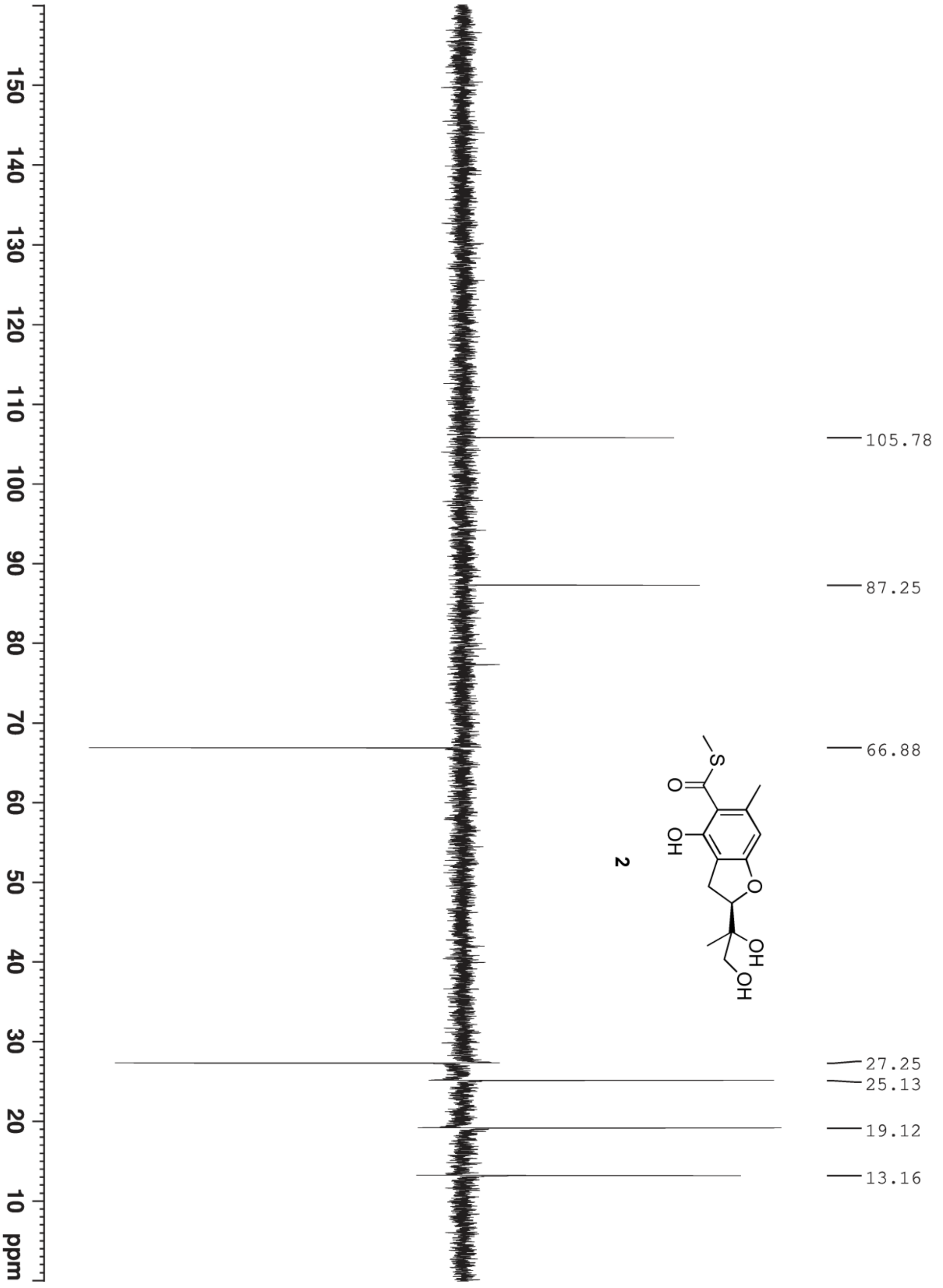

Figure S12. ^{1}H–^{1}H COSY spectrum of eurothiocin B (**2**).

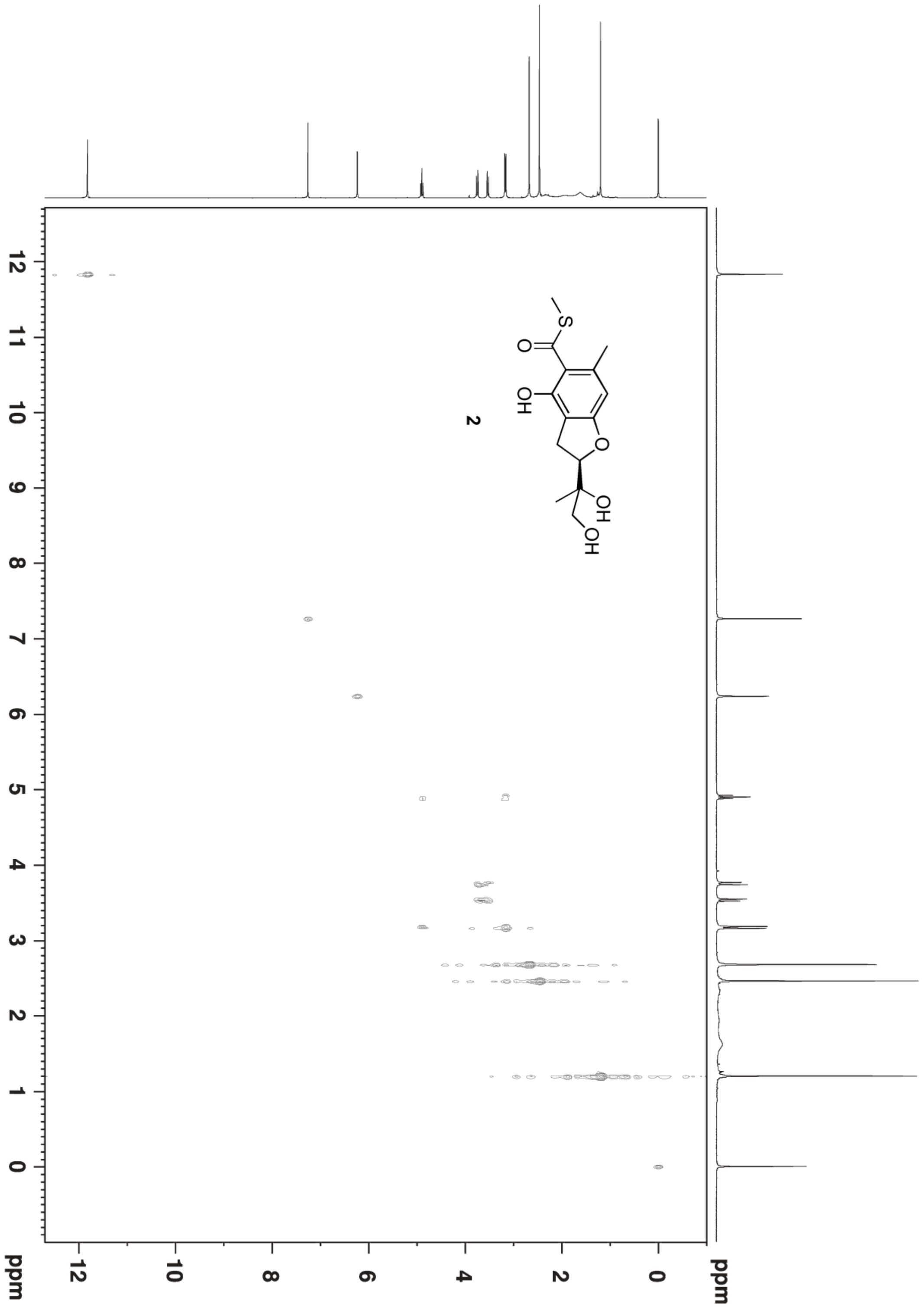

Figure S13. HSQC spectrum of eurothiocin B (**2**).

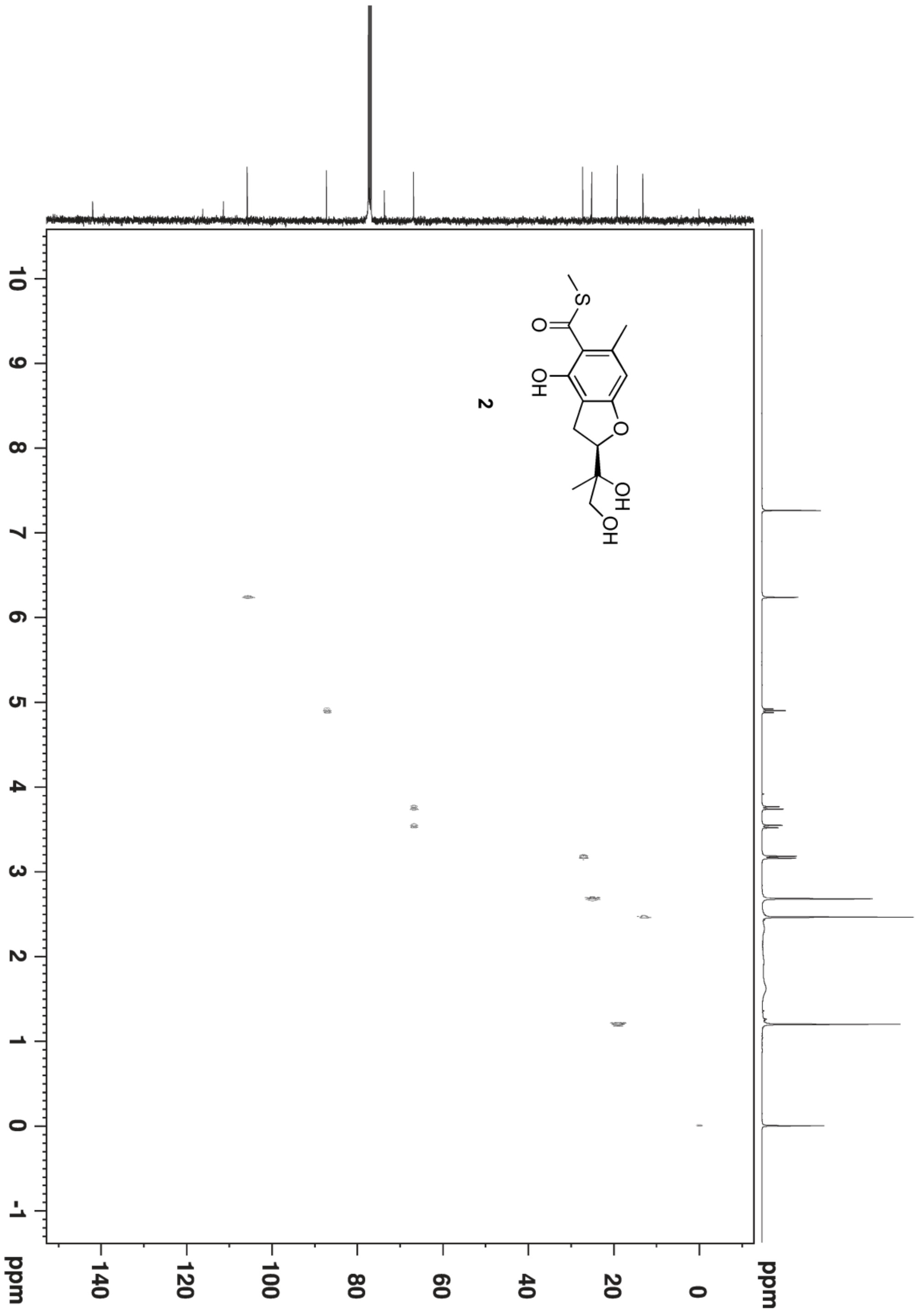

Figure S14. HMBC spectrum of eurothiocin B (**2**).

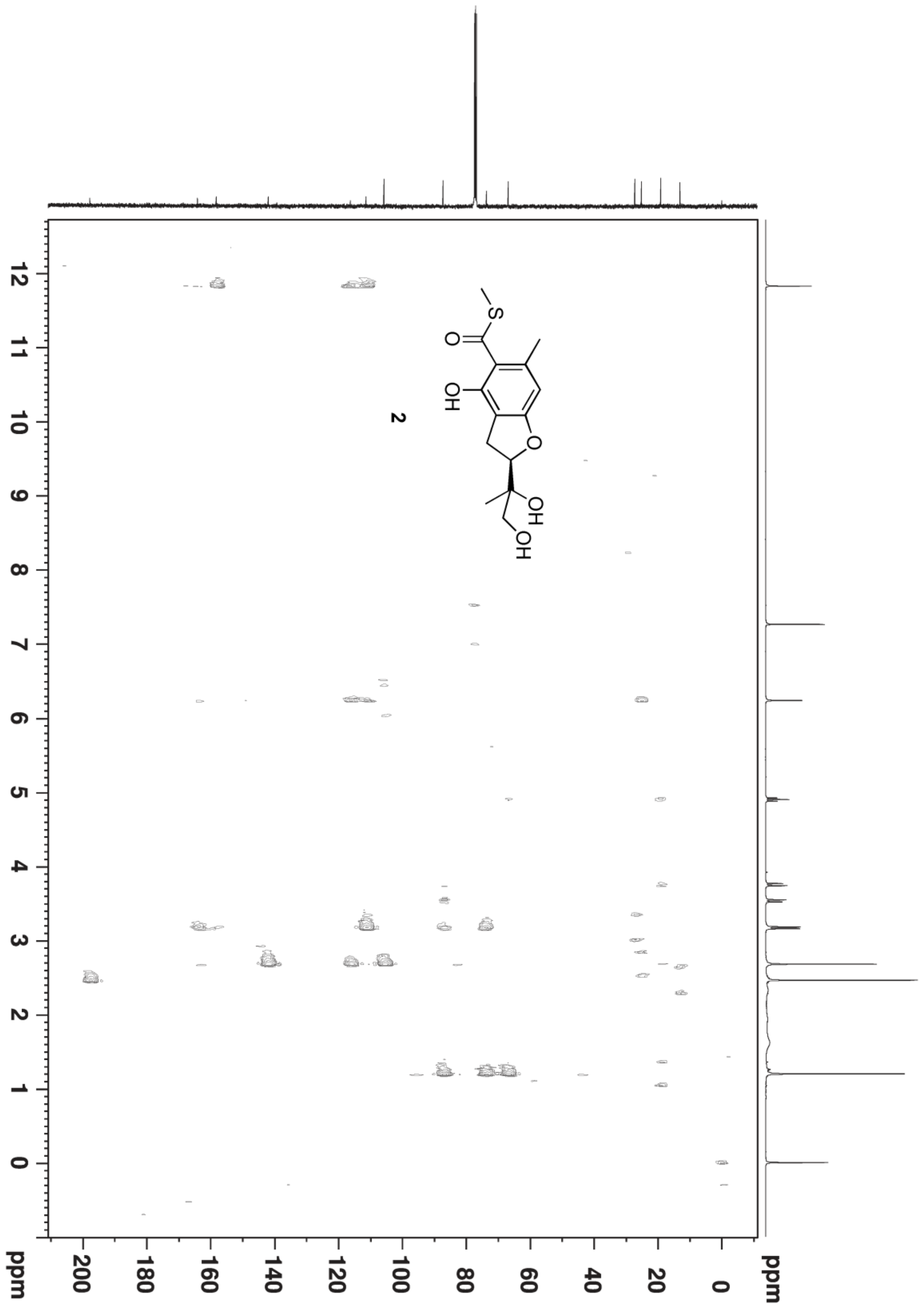

Figure S15. Calculated ECD spectra of the four most stable conformers of (*R*)-**1**.

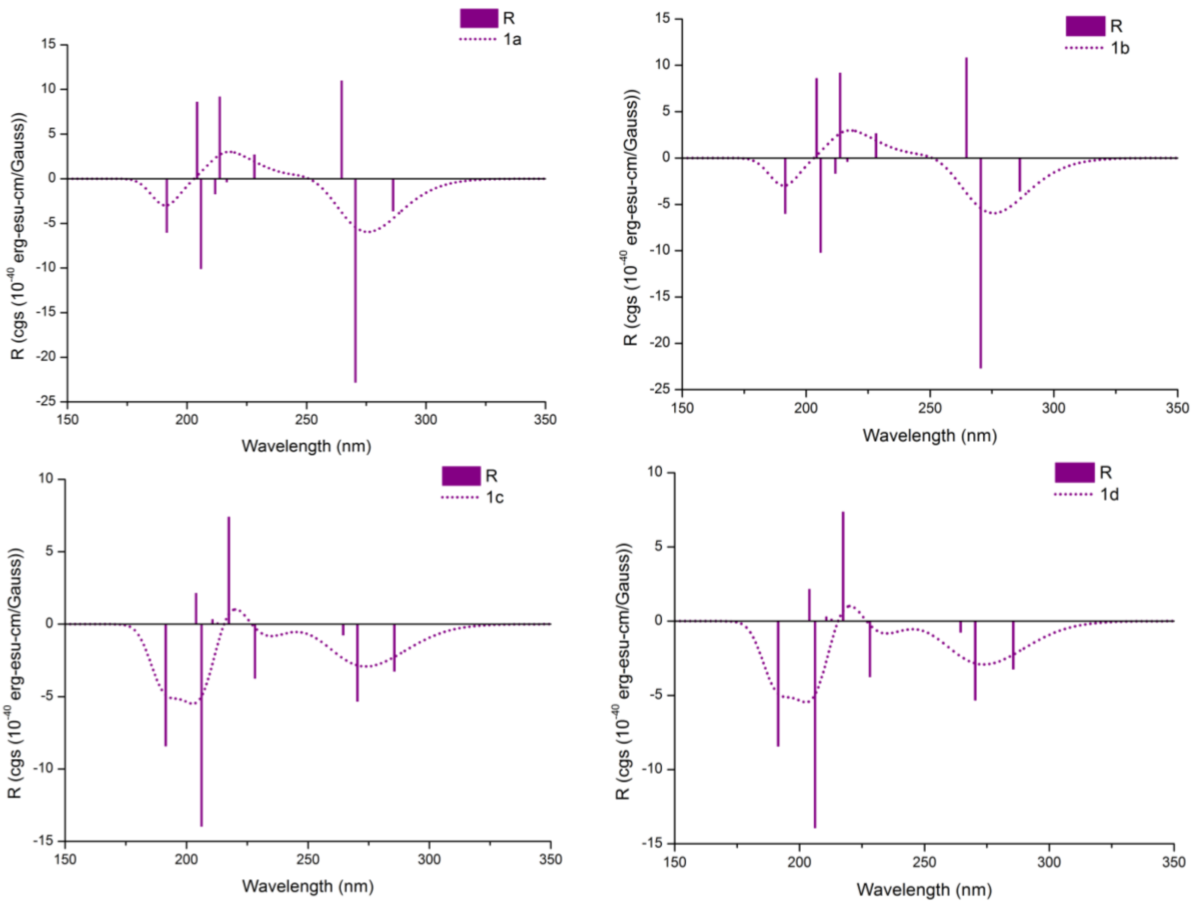

© 2014 by the authors; licensee MDPI, Basel, Switzerland. This article is an open access article distributed under the terms and conditions of the Creative Commons Attribution license (http://creativecommons.org/licenses/by/3.0/).
